# Interhemispheric–Transcortical Approach for Resection of an Atypical Teratoid/Rhabdoid Tumor (AT/RT) of the Left Lateral and Third Ventricle

**DOI:** 10.1055/a-2701-4192

**Published:** 2025-09-30

**Authors:** Douglas Chung, Patrick F. O' Brien, Hasan Syed

**Affiliations:** 1 Department of Neurological Surgery, George Washington University, Washington, District of Columbia, United States; 2 Department of Neurological Surgery, Children's National Medical Center, Washington, District of Columbia, United States

**Keywords:** AT/RT, atypical teratoid/rhabdoid tumor, ventricular surgery, pediatric neuro-oncology, high-grade glioma

## Abstract

**Introduction:**

Atypical teratoid/rhabdoid tumors (AT/RT) are malignant central nervous system (CNS) tumors that represent 3% of all pediatric CNS tumors. Majority of cases have SMARCB1 gene mutations and historically carried a poor prognosis.

**Case Presentation:**

A 9-year-old boy was diagnosed with a third ventricle AT/RT and initially underwent endoscopic surgical biopsy. Subsequent tumor resection was completed using an interhemispheric–transcortical approach.

**Lessons:**

Surgical approaches to ventricular system must be tailored to the tumor characteristics on a case-by-case basis. We present a case using a transcortical approach for understanding of the microsurgical anatomy for safe resection of a third ventricle AT/RT.

## Transcript ( Video 1; mm:ss )

**Video 1** Operative video demonstrating an interhemispheric-transcortical approach for resection of a third ventricle atypical teratoid/rhabdoid tumor (AT/RT) extending into the left lateral ventricle.


[0:00] Title Page

This video demonstrates an interhemispheric–transcortical approach for resection of a third ventricle atypical teratoid/rhabdoid tumor (AT/RT) extending into the left lateral ventricle.

[0:10] Clinical Presentation

The patient is a 9-year-old previously healthy boy who presented with several weeks of progressive gait instability and worsening headache.

[0:18] Preoperative MRI, description of clinical course

An MRI demonstrates a heterogeneously enhancing mass occupying the third ventricle with extension into the left lateral ventricle causing obstructive hydrocephalus. The patient initially underwent a left frontal endoscopic biopsy and EVD placement. 1 The prelim pathology initially came back as high-grade glioma. After a multi-disciplinary discussion with the patient and his family, he returned to the OR for tumor resection.

[0:45] Surgical Strategy

An interhemispheric craniotomy was planned with the patient positioned supine. The surgical strategy consists of a transcortical approach utilizing the prior EVD tract to enter the left lateral ventricle with identification of the tumor. 2 Identification of the thalamostriate vein and choroid plexus helps us notice a medially displaced foramen of Monro, followed by tumor debulking which would demonstrate the extension through the floor of the lateral ventricle, and complete tumor resection within the third ventricle with identification of the tuber cinereum and underlying basilar artery. 3 This was followed by a septum pellucidotomy and third ventriculostomy for additional CSF diversion. 4

[1:26] Intraoperative photo for orientation

Here we have an intraoperative view of the left lateral ventricle with the right side of the screen medial to the patient, the left side of the screen lateral, and the top of the screen is anterior with the tumor visualized in the center of the screen.

[1:39] Demonstrates tumor in lateral ventricle, which is easily suckable

Again we have the tumor visualized within the left lateral ventricle, which appears red, vascular, and soft. This demonstrates that the tumor is soft and easily suckable with the suction tip.

[1:59] Identification of the thalamostriate vein, choroid plexus

With careful tumor debulking within the left lateral ventricle, the thalamostriate vein and choroid plexus are identified in the ventricular floor.

[2:01] Example of normal surgical anatomy of lateral ventricle

This is a view of the normal anatomy of the lateral ventricle, where the thalamostriate vein and choroid plexus can be followed ventral to the foramen of Monro. 3

[2:11] Foramen of Monro is small, displaced medially by tumor

Further exposure of the thalamostriate vein and choroid plexus eventually leads us to a medially displaced foramen of Monro and tumor which appears to be extending through the floor of the lateral ventricle.

[2:25] Annotated still image showing foramen of Monro, thalamostriate/septal veins, choroid plexus

[2:29] Tumor resection into 3rd ventricle; preliminary pathology initially showed anaplastic ependymoma, therefore gross total resection was pursued

The tumor resection was continued into the third ventricle with identification of the third ventricular walls. The preliminary pathology was consistent with an anaplastic ependymoma and therefore, gross-total resection was pursued. The third ventricle can be seen with gross-total resection of the tumor.

[2:49] Identification of tuber cinereum, creation of third ventriculostomy with identification of basilar artery underneath

At the third ventricular floor, the tuber cinereum as well as an underlying basilar artery was identified, and a third ventriculostomy was performed. Here the third ventriculostomy is gently dilated and we can clearly see the underlying basilar artery.

[3:07] Creation of septum pellucidotomy

A septum pellucidotomy was created and further dilated for additional CSF diversion.

[3:14] Post-operative MRI

Postoperative MRI demonstrated gross-total resection of the tumor and resolution of the obstructive hydrocephalus. There were no new neurological deficits postoperatively. The patient was monitored in the pediatric ICU and treated for diabetes insipidus postoperatively, which resolved spontaneously with supportive care. The patient failed an EVD wean trial and on postoperative day 8, underwent a right-sided ventriculoperitoneal shunt placement with a non-programmable valve. He was discharged home with family on postoperative day 9.

[3:56] Outcome

An outpatient lumbar puncture was negative for malignant cells on CSF cytology. A 2-week surveillance MRI was obtained, which demonstrated post-surgical changes and a small spot of residual enhancement in the anterior third ventricle. The patient was started on focal radiation therapy for initially presumed anaplastic ependymoma. However, the final pathology revealed biallelic deletion of SMARCB1, confirming a diagnosis of AT/RT, and the patient was transitioned to undergo craniospinal radiation. 5 At 1-month follow-up, the patient continued to do well and will follow up with neuro-oncology to discuss options for adjuvant chemotherapy.

[4:19] AT/RT are malignant pediatric central nervous system tumors, with majority of cases carrying SMARCB1 gene mutations and have historically had poor prognosis. 5 Management typically includes maximal safe resection with tissue diagnosis, either focal or craniospinal radiation, and chemotherapy. 5 Additionally, there are many ongoing clinical trials investigating targeted therapies or immunotherapies as further adjuvants to treatment. 5

[4:46] In summary, we present a case of a third ventricular tumor with extension into the left lateral ventricle, initially thought to be an anaplastic ependymoma but later confirmed to be AT/RT, that was safely resected using an interhemispheric–transcortical approach.
